# A new strategy for enhancing imputation quality of rare variants from next-generation sequencing data via combining SNP and exome chip data

**DOI:** 10.1186/s12864-015-2192-y

**Published:** 2015-12-29

**Authors:** Young Jin Kim, Juyoung Lee, Bong-Jo Kim, T2D-Genes Consortium, Taesung Park

**Affiliations:** Interdisciplinary Program in Bioinformatics, Seoul National University, Seoul, 151-742 South Korea; Division of Structural and Functional Genomics, Center for Genome Science, Korean National Institute of Health, Osong, Chungchungbuk-do 363-951 South Korea; Department of Statistics, Seoul National University, San 56-1, Shilim-dong, Kwanak-gu, Seoul 151-742 South Korea

**Keywords:** Combined approach, Exome chip, Imputation, Rare variant

## Abstract

**Background:**

Rare variants have gathered increasing attention as a possible alternative source of missing heritability. Since next generation sequencing technology is not yet cost-effective for large-scale genomic studies, a widely used alternative approach is imputation. However, the imputation approach may be limited by the low accuracy of the imputed rare variants. To improve imputation accuracy of rare variants, various approaches have been suggested, including increasing the sample size of the reference panel, using sequencing data from study-specific samples (i.e., specific populations), and using local reference panels by genotyping or sequencing a subset of study samples. While these approaches mainly utilize reference panels, imputation accuracy of rare variants can also be increased by using exome chips containing rare variants. The exome chip contains 250 K rare variants selected from the discovered variants of about 12,000 sequenced samples. If exome chip data are available for previously genotyped samples, the combined approach using a genotype panel of merged data, including exome chips and SNP chips, should increase the imputation accuracy of rare variants.

**Results:**

In this study, we describe a combined imputation which uses both exome chip and SNP chip data simultaneously as a genotype panel. The effectiveness and performance of the combined approach was demonstrated using a reference panel of 848 samples constructed using exome sequencing data from the T2D-GENES consortium and 5,349 sample genotype panels consisting of an exome chip and SNP chip. As a result, the combined approach increased imputation quality up to 11 %, and genomic coverage for rare variants up to 117.7 % (MAF < 1 %), compared to imputation using the SNP chip alone. Also, we investigated the systematic effect of reference panels on imputation quality using five reference panels and three genotype panels. The best performing approach was the combination of the study specific reference panel and the genotype panel of combined data.

**Conclusions:**

Our study demonstrates that combined datasets, including SNP chips and exome chips, enhances both the imputation quality and genomic coverage of rare variants.

## Background

Genome-wide association studies (GWAS) have revealed unprecedented numbers of disease-associated loci [1, 2]. However, previously reported loci explain only a small proportion of heritability [2–4]. Previous GWAS mainly focused on common variants that was readily accessible via initial genomic technologies [2]. Through recent advancements in high-throughput sequencing technology, more complete genome-wide assessment of variants has become possible [5]. Recent large scale sequencing studies reported that the population frequencies of a large proportion of discovered variants were rare (Minor Allele Frequency (MAF) < 1 %) [6–8]. Given their abundance, rare variants have been increasingly recognized as an alternative source of missing heritability [5, 7, 9]. However, large-scale, population-based genomic sequencing studies are not yet feasible, due to high cost and computation-intensive analysis [10, 11].

Alternatively, imputation has been widely used for studying rare variants. Imputation has estimated untyped rare variants using thousands of sequenced samples available as a reference panel such as the 1,000 genomes project data [12, 13]. Recent imputation-based association studies have revealed numerous uncommon or rare variants associated with coronary artery disease, blood cell traits, serum creatinine, chronic kidney disease, and adult body height [10, 12, 13]. However, imputing rare variants has been challenging, due to the low accuracy of imputed genotypes of rare variants [10, 14], and poorly imputed rare variants may mislead follow-up association studies.

Imputation requires a reference panel and genotype panels. The reference panel is the basis for imputation performance, and the genotype panel is made from observed data. From both the reference and genotype panels, the shared haplotype segments are estimated using variants present on both panels. Then, the untyped genotypes are imputed using these haplotypes [15]. The accuracy of imputation can be increased by improving the reference panel and the genotype panel.

Previously, numerous studies have reported enhanced imputation performance of rare variants [14, 16–19], using three types of basic approaches for improving reference panels. The first is to increase the number of samples of the reference panel [14]. The second approach uses a study-specific reference panel instead of a public reference panel (e.g., the 1,000 genomes project reference panel) [16, 17]. The last ‘two step approach’ uses a local reference panel consisting of a subset of samples with a chip containing many low frequency variants or local sequencing data [18, 19]. Such local reference panels are used to complement public reference panels. These three approaches mainly focus on improving the reference panels by constructing an independent, study-specific reference panel, or complementing an existing public reference panel.

Alternatively, the imputation accuracy of rare variants can also be increased by improving a genotype panel using a chip designed to contain rare variants or markers tagging nearby rare variants [14, 18]. For example, an exome chip is a customized chip containing about 250 K variants including rare functional coding variants selected from ~ 12,000 sequenced samples [20] that can be genotyped at less cost than commercial genome-wide single nucleotide polymorphism (SNP) chips containing rare variants. While exome chips alone were shown as insufficient for imputation, as compared to commercial SNP chips widely used for GWAS [21], exome chips additionally genotyped for previously SNP chip-genotyped samples would improve their utility as a good source of rare variants. Moreover, a genotype panel combining exome chips and SNP chip data can help construct population-specific haplotypes carrying rare variants, thus also increasing the imputation accuracy of rare variants.

In this study, we propose a new strategy to increase the accuracy of imputation of rare variants by improving a new genotype panel by combining exome chip with existing SNP chip data. We show that the new genotype panel of combined data of exome chip and SNP chip improves imputation quality of imputed rare variants. To demonstrate the effectiveness of our strategy for improving genotype panels, we compared imputation strategies based on three genotype panels: 1) exome chip only; 2) SNP chip only; and 3) combined SNP chip and exome chip. Performances were compared via imputation quality scores [22] and genomic coverages [23, 24].

We also performed a systematic investigation of the effect of the reference panel on the imputation quality of rare variants. Using 848 samples with whole exome sequencing data (WES), SNP chip data (GWAS), and exome chip data (EXOME), we built various reference panels: WES + GWAS + EXOME, WES + GWAS, WES + EXOME, WES, and the 1000 genomes phase 1 dataset (1KG). We then performed imputation on 5,349 samples with three genotype panels of exome chip, SNP chip, and combined data. Additionally, to assess the effect of the reference panel sample size on imputation performance, we varied the number of samples from 300 to 848, by increments of 200, to examine the performance of imputation strategies.

## Results

### Exclusion of poorly-imputable variants

In this study, we used the estimated *r*^2^ (${\widehat{r}}^2$) values provided by the imputation software minimac [15, 25] as imputation quality scores. The ${\widehat{r}}^2$ is an estimate of the true *r*^2^ (dosage *r*^2^), which is the squared correlation of the true genotypes and imputed genotypes [25]. It is given by comparing the variance of imputed genotype scores with the variance of expected genotype scores. Previously, Li et al. reported that ${\widehat{r}}^2$ was poorly estimated for very rare variants (MAF ≤ 0.5 %) [14]. Almost all variants with poorly calibrated ${\widehat{r}}^2$ were very rare variants. Also, the Pearson correlation coefficient between ${\widehat{r}}^2$ and dosage *r*^2^ was below 0.9 (0.783–0.825) for very rare variants, but it was more than 0.9 (0.951–0.983) for variants with MAF > 0.5 % [14]. If the ${\widehat{r}}^2$ could not estimate the true value closely, then our comparative analysis of imputation performance would yield misleading results. In this context, we called very rare variants as “poorly-imputable variants” if the Pearson correlation coefficient ${\widehat{r}}^2$ and dosage *r*^2^ was below 0.9 and excluded them for further analysis.

**Fig. 1:**
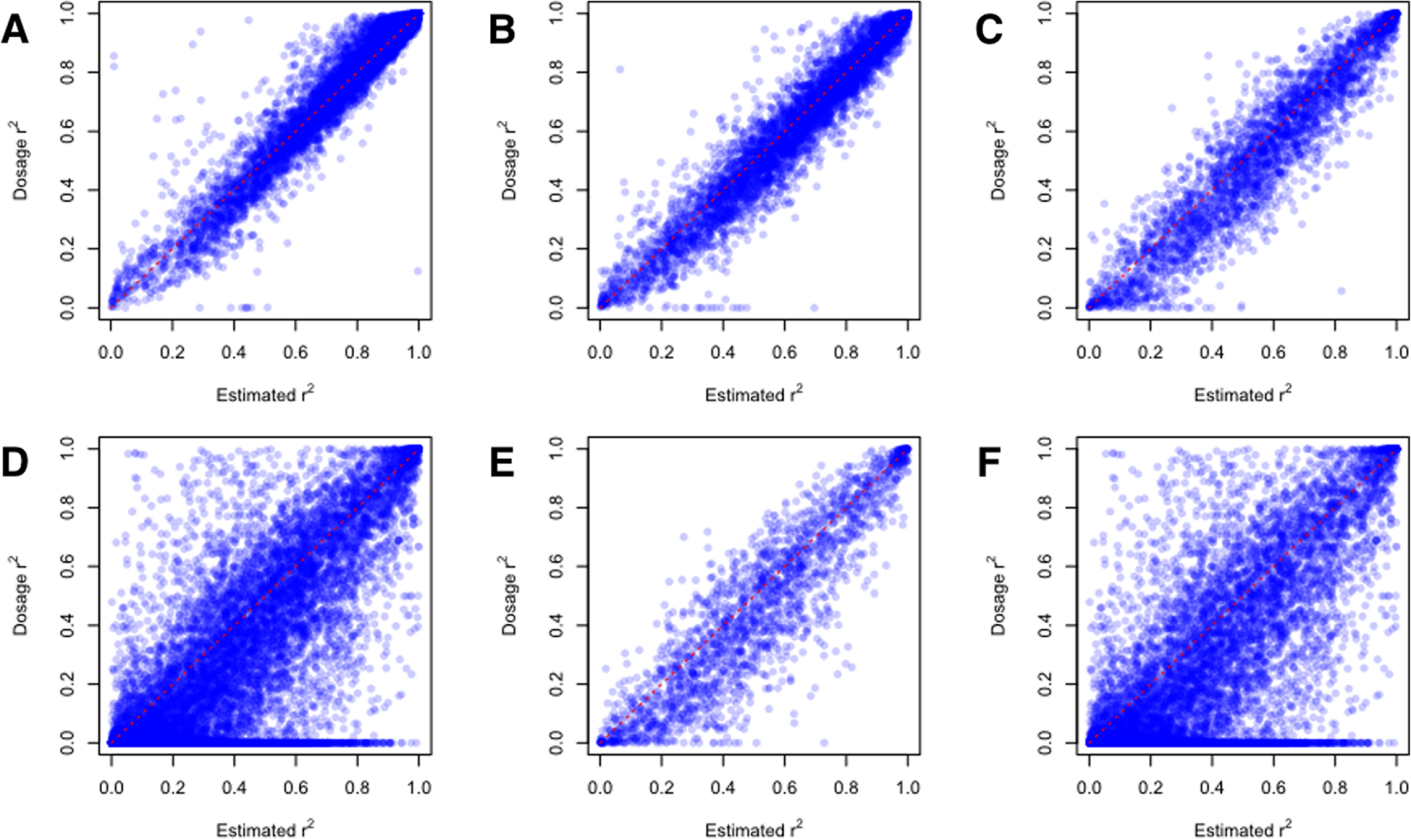
Scatter plot of estimated r^2^ against dosage r^2^ by MAF bins. Estimated r^2^ was plotted against dosage r^2^ by MAF bins (**a**) MAF ≥ 5 %, (**b**) MAF = 1–5 %, (**c**) MAF = 0.5–1 %, (**d**) MAF < 0.5 %, (**e**) MAF = 0.3–0.5 %, and (**f**) MAF < 0.3 %. The red dotted line represents the diagonal

We compared ${\widehat{r}}^2$ and dosage *r*^2^ in four different MAF bins. We divided the variants into four MAF bins based on MAF: common (MAF ≥ 5 %), less common (MAF 1–5 %), rare (MAF 0.5–1 %), and very rare (MAF < 0.5 %) [14]. To measure the strength of the linear relationship between ${\widehat{r}}^2$ and dosage *r*^2^, Pearson correlation coefficients were calculated for each MAF bin. We first performed imputation on the genotype panel containing the SNP chip only by using our WES + GWAS + EXOME reference panel. Among imputed variants, 45,802 variants from 5,349 samples were compared to the corresponding variants obtained from an exome chip constructed using identical samples. Figure 1 shows the imputation results of variants by MAF bins. The Pearson correlation coefficients were 0.98, 0.97, 0.94, and 0.77 for MAF bins ≥5 %, 1–5 %, 0.5–1 %, and < 0.5 %, respectively. As Li et al. reported, ${\widehat{r}}^2$ did not reflect the true value, dosage *r*^2^, for very rare variants (MAF < 0.5 %, Fig. 1d). However, ${\widehat{r}}^2$ became closer to the dosage *r*^2^, as the MAF increased (Fig. 1a–c).

In this study, we used whole exome sequencing data (~18,000 genes) for studying imputation performance. However, Li et al. studied imputation performance using sequencing data of only 202 genes [14]. Since the results may be different depending on the data, we thoroughly analyzed very rare variants to study the lower bound of allele frequency showing good estimation of dosage *r*^2^. Very rare variants were split into discrete MAF bins of width 0.001. The Pearson correlation coefficients were 0.91, 0.90, 0.87, 0.78, and 0.55 for MAF bins 0.4–0.5 %, 0.3–0.4 %, 0.2–0.3 %, 0.1–0.2 %, and 0–0.1 %, respectively. The Pearson correlation coefficients were 0.98, 0.97, 0.94, 0.77 for MAF bins ≥ 5 %, 1–5 %, 0.5-1 %, and < 0.5 %, respectively. Among very rare variants, MAF bins with MAF < 0.3 % showed that the Pearson correlation coefficients dropped below 0.9 (Fig. 1e–f). If these variants are included in the analysis, poorly estimated ${\widehat{r}}^2$ may cause less consistent results to those using dosage *r*^2^. Therefore, the variants with MAF < 0.3 % (369,309 of 856,690 variants) were regarded as poorly-imputable and excluded from further study.

### Comparison of imputation quality among genotype panels

**Fig. 2:**
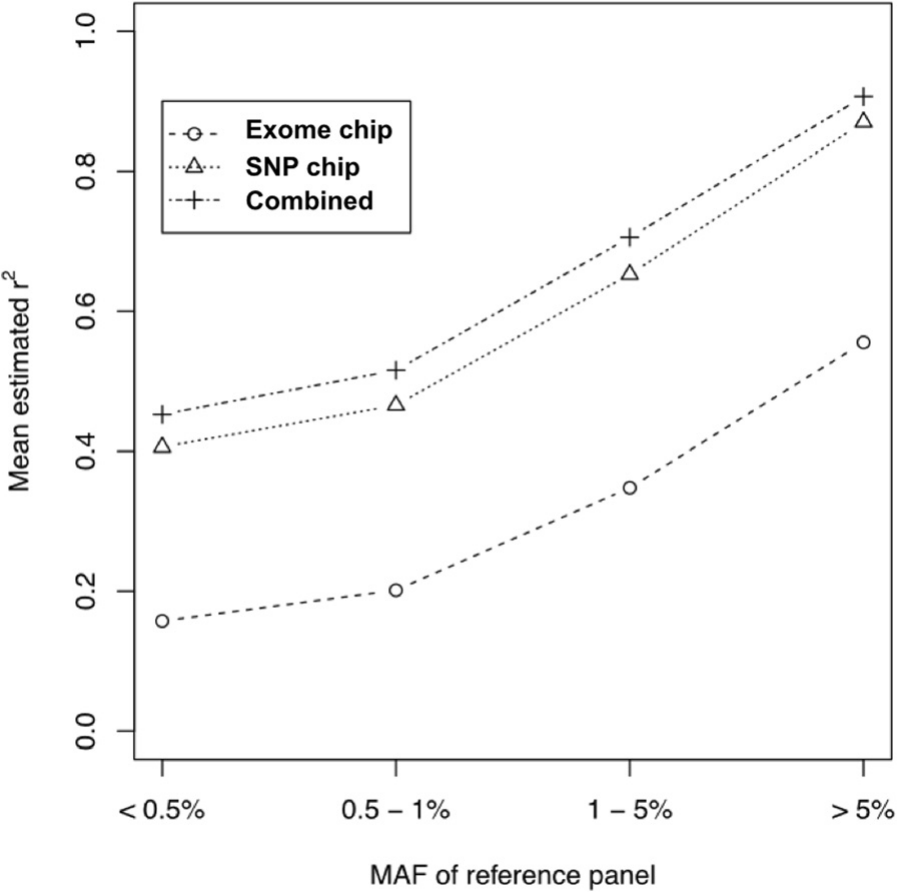
Mean estimated r^2^ of genotype panels by MAF bins

Using the WES + GWAS + EXOME reference panel excluding poorly imputable variants, we performed imputation on three genotype panels, including exome chip only, SNP chip only, and combined data of exome chip and SNP chip. For the purpose of comparison, we selected 108,682 imputed variants overlapping the three genotype panels. The ${\widehat{r}}^2$ value was used to measure imputation quality. The numbers of variants were 35,443 (32.6 %), 21,191 (19.5 %), 19,527 (18.0 %), and 32,547 (29.9 %) for variants with MAF ≥ 5 %, 1−5 %, 0.5−1 %, and < 0.5 %, respectively. Figure 2 shows the comparison results. As previously reported, the genotype panel of the exome chip alone showed the worst performance [21]. The mean ${\widehat{r}}^2$ values were 0.332, 0.616, and 0.661 for the genotype panels of exome chip, SNP chip, and combined approach, respectively. Thus, the combined genotype panel showed the best performance compared to the other genotype panels (Wilcoxon signed rank sum test *P*-values < 2.2x10^−16^, a 7.3 % relative increase in the mean of the ${\widehat{r}}^2$ compared to those of SNP chip only). In Fig. 2, most imputed variants using the combined approach performed better than the genotype panel of SNP chip alone. The mean values of the ${\widehat{r}}^2$ of SNP and combined approach were 0.870 and 0.906 for MAF ≥ 5 %, 0.653 and 0.706 for MAF 1−5 %, 0.465 and 0.515 for MAF 0.5−1 %, and 0.406 and 0.452 for MAF <0.5 %, respectively. The relative increment in ${\widehat{r}}^2$ of the combined genotype panel was 4.1 % for common variants (MAF ≥ 5 %), 8.1 % for less common variants (MAF 1−5 %), 10.7 % for rare variants (MAF 0.5−1 %), and 11.4 % for very rare variants (MAF < 0.5 %), compared to the genotype panel with SNP chip only. Thus, the increment in imputation quality was largest when the minor allele frequencies of the imputed variants were below 1 %.

### Comparison of genomic coverage among genotype panels

We next compared the genotype panels in terms of their genomic coverage, i.e., the proportion of variants captured by a genotyping chip [24]. The larger the genomic coverage, the better the association mapping performance. One major advantage of imputation lies in obtaining a large dense set of imputed variants from a relatively small number of observed variants on the genotype panel. These imputed variants enhanced genomic coverage, resulting in increased association power [15], enabling us to perform *in silico* fine mapping in imputation-based association studies. Likewise, Nelson et al. recently reported imputation-based genomic coverage of widely used genotyping chips [24]. Imputation-based genomic coverage is calculated as the number of imputed variants with quality scores above the threshold value (info score ≥ 0.8) divided by the total number of variants in the reference panel [24].

In this study, we compared imputation-based genomic coverage of three genotype panels. We imputed genotype panels using the WES + GWAS + EXOME reference panel. For genomic coverage, we selected 143,022 exonic variants, including those imputed and genotyped by exome chip. Since we used exome sequencing data to construct the reference panel, 143,022 variants were regarded as a “virtual” exome. The numbers of variants were 56,326 (39.4 %), 28,072 (19.6 %), 22,931 (16.0 %), and 35,693 (25.0 %) with MAFs ≥ 5 %, 1−5 %, 0.5−1 %, and < 0.5 %, respectively. Table 1 summarizes the genomic coverages. We also selected two cut-off values for ${\widehat{r}}^2$: 0.8 as a stringent cut-off, and 0.4 as a less stringent cut-off. This 0.8 stringent cut-off provided a genomic coverage of 0.435 for the SNP chip only and 0.560 for the combined approach, respectively, while the less stringent cut-off (${\widehat{r}}^2$≥ 0.4) provided genomic coverages of 0.749 and 0.818 for SNP chip only and the combined approach, respectively. Overall, the combined approach yielded approximately 9.2 % (${\widehat{r}}^2$ ≥ 0.4) and 29 % (${\widehat{r}}^2$≥ 0.8) relative increases in genomic coverage of all variants. However, for rare variants (MAF < 1 %) applying stringent cut-offs (${\widehat{r}}^2$ ≥ 0.8), the genomic coverage of the combined approach increased by 98.6 % and 117.7 %, compared to that of the SNP chip only, for variants with MAFs of 0.5–1 % and < 0.5 %, respectively. Thus, the combined approach greatly improved genomic coverages of rare variants.

**Table 1:** Genomic coverage of genotype panels of SNP chip only and combined approach

MAF bin	Estimated* r* ^2^ ≥ 0.8			Estimated* r* ^2^ ≥ 0.4		
	Exome chip	SNP chip	Combined	Exome chip	SNP chip	Combined
ALL	0.367	0.435	0.560	0.492	0.749	0.818
≥5 %	0.600	0.794	0.901	0.756	0.953	0.983
1–5 %	0.374	0.403	0.588	0.510	0.799	0.881
0.5–1 %	0.192	0.146	0.290	0.290	0.585	0.686
<0.5 %	0.107	0.079	0.172	0.192	0.491	0.591

### Systematic effect of various reference panels on imputation quality

**Fig. 3:**
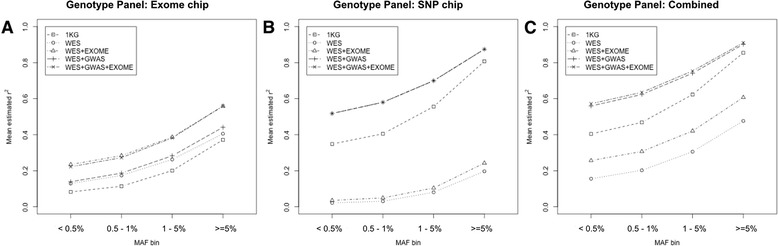
Mean estimated *r* ^2^ of various combinations of reference panels and genotype panels. Reference panels are the 1000 genomes phase 1 dataset (1KG) and various combinations of whole exome sequencing data (WES), SNP chip data (GWAS), and exome chip data (EXOME)

Systematic analysis of reference panels could establish an effective strategy for improving imputation quality of rare variants. We first investigated systematically the effects of various reference panels on imputation quality using five different reference panels, including 1KG, WES, WES + EXOME, WES + GWAS, and WES + GWAS+ EXOME. We then compared imputation qualities by using 15 possible combinations of three genotype panels and five reference panels. ${\widehat{r}}^2$ values were used to measure imputation quality. For comparisons, we selected 66,920 variants imputed by all 15 combinations. Figure 3 shows the imputation qualities by genotype panels used: Fig. 3a for exome chip, 3b for SNP chip, and 3c for the combined. Imputation quality was influenced by reference panels, and the same reference panel showed different imputation quality when used for imputing different genotype panels. The WES + EXOME reference panel was the best performing genotype panel of exome chips, and the WES + GWAS + EXOME reference panel was best for the genotype panels of SNP chip and the combined data. The 1KG reference panel was the worst performing genotype panel of exome chip, and the WES reference panel for the genotype panels of SNP chip and the combined data.

We next investigated why imputation quality differed across the reference panels, and found two factors: 1) differences in the number of overlapping variants between the reference and genotype panels; and 2) shared haplotype patterns between the reference and genotype panels.

First, overlapping variants between the reference and genotype panels play important roles in imputation. For predicting untyped genotypes, imputation uses estimated haplotype segments from the reference panel for the overlapping variants between reference and genotype panels. The lower the number of overlapping variants between the reference and genotype panels, the worse the imputation quality, due to inaccurate estimation of haplotype segments. The numbers of overlapping variants are summarized in Table 2. For study-specific reference panels that included WES data, the worst performing reference panel had the smallest number of overlapping variants, while the best performing reference panel had the most overlapping variants. This is the reason why the best and worst performing reference panels were different according to the genotype panels used. For example, WES + EXOME reference panel showed the best performance for the genotype panel of exome chip (Fig. 3a). The number of overlapped variants was 38,243, which was the largest number among the number of overlapping variants between the four study-specific reference panels and genotype panel of exome chip (Table 2). On the other hand, WES + EXOME was the 2^rd^ worst performing for other genotype panel, having the 2^nd^ smallest number of overlapping variants (Figs. 3a and 3b, Table 2).

**Table 2:** The number of overlapped variants between reference panels and genotype panels

Reference panels	Exome chip	SNP chip	Combined
WES	21,120	4,472	24,514
WES + EXOME	38,243	7,323	41,637
WES + GWAS	23,972	344,359	364,402
WES + GWAS + EXOME	38,243	344,359	378,695
1KG	49,286	344,359	389,715

Second, the use of shared haplotype patterns between the reference and genotype panels also improved the accuracy of imputation. Although the 1KG reference panel had the largest number of overlapped variants, its imputation quality was worse than using study-specific reference panels such as WES + EXOME for exome chip (Fig. 3a) and WES + GWAS + EXOME for the genotype panels of SNP chip and the combined data (Fig. 3b and 3c). As previously reported [17], the better performance of study-specific reference panels over 1KG was due to more shared haplotype segments between study-specific reference panels and the genotype panels used [17, 26]. All samples of study-specific reference panels and genotype panels were from a Korean population, whereas the 1KG reference panel consists of samples from various populations such as Europeans, Africans, and Asians. Thus, the 1KG reference panel and the genotype panel of Korean population do not have many shared haplotypes, resulting in poor imputation.

Comparative analysis of the 15 different combinations provided us effective imputation strategies for imputing rare variants when a specific genotype panel is given with a specific reference panel. For example, compared to the case of the SNP chip genotype panel with the 1KG reference panel, three strategies could increase the imputation quality of rare variants: 1) use the best performing reference panel, e.g., WES + GWAS + EXOME, instead of 1KG; 2) construct the genotype panels of combined data by additional genotyping of an exome chip for those samples already genotyped using the SNP chip; and 3) use the genotype panel of combined data from (2, above) with WES + GWAS + EXOME as a reference panel. The first strategy increased imputation quality from 0.445 to 0.622 for rare variants and from 0.377 to 0.553 for very rare variants. When the second strategy was applied, the imputation quality was 0.503 and 0.426 for rare and very rare variants, while the best performing strategy was the third, increasing imputation quality to 0.664 and 0.595 for rare and very rare variants, respectively.

### Sample size effect of reference panel on imputation quality

**Fig. 4:**
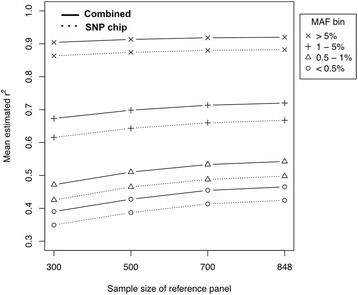
Mean estimated r^2^ varied by sample size of reference panel

It was previously shown that the larger the sample size of the reference panel, the better the imputation quality [14]. Here, we systematically investigated the effect of sample size on imputation quality. Since the WES + GWAS + EXOME reference panel performed the best, we studied the sample size effect of WES + GWAS + EXOME reference panel on imputation quality. We performed imputation on the genotype panels of SNP chip and combined data with reference sample sizes of 300, 500, 700, and 848. This analysis was restricted to variants on chromosome 1, to focus only on overlapping imputed variants. The total number of imputed variants used for this analysis was 10,624. Figure 4 shows the mean ${\widehat{r}}^2$ of SNP chip only and combined data by different sample sizes for the reference panel. As expected, imputation quality increased as the sample size of reference panel increased.

For the imputed rare variants (MAF 0.5–1 %) using the combined data genotype panel, relative increases in mean ${\widehat{r}}^2$ values were 8.2 % and 4.5 % for increasing the reference panel sample size from 300 to 500, and 500 to 700, respectively (Table 3), and 0.93 % for sample size increases from 700 to 848. Increments in mean ${\widehat{r}}^2$ values were the lowest between 700 and 848 samples used for the reference panel. Therefore, increasing the sample size to more than 700 would not be cost-effective for improving imputation quality of rare variants. Similarly, imputation qualities of very rare variants (MAF <0.5 %) showed similar patterns to those of rare variants. When we performed the same analysis for the SNP chip genotype panel, similar patterns were observed for both rare and very rare variants.

**Table 3:** Relative increase in mean estimated *r* ^2^ by reference sample size (MAF 0.5–1 %)

Genotype panel	300 to 500	500 to 700	700 to 848
SNP chip only	9.27 %	4.90 %	2.09 %
Combined (SNP + exome chip)	8.18 %	4.46 %	1.74 %

This study of sample size also provided us cost-effective sample size determination strategies for imputing rare variants. Instead of constructing a reference panel with a large number of samples, it would be more cost-effective to combine the reference panel with a smaller number of samples and the genotype panel of combined data. Table 4 (MAF 0.5–1 %) summarizes the relative increases in imputation quality. For example, the genotype panel of combined data with 500 samples of reference panel showed better imputation quality (mean ${\widehat{r}}^2$ = 0.510) than the genotype panel of SNP chip only data with 848 samples of reference panel (mean ${\widehat{r}}^2$ = 0.498) (Fig. 4).

**Table 4:** Relative increase in mean estimated *r* ^2^ by using combination of reference panel and combined approach (MAFs 0.5–1 %)

	R300-C	R500-C	R700-C	R848-C
R300-G	10.88 %	19.95 %	25.30 %	27.49 %
R500-G	1.47 %	9.77 %	14.67 %	16.67 %
R700-G	−3.27 %	4.64 %	9.31 %	11.21 %
R848-G	−5.25 %	2.50 %	7.07 %	8.94 %

The names of panels were abbreviated as follows: R (reference panel), G (the genotype panel of SNP chip only), and C (the genotype panel of combined data). R300, R500, R700, and R848 indicates sample sizes of 300, 500, 700, and 848 for reference panel, respectively. R(sample size) -G represents combination of R(sample size) reference panel and G genotype panel. R(sample size)-C represents the combination of R(sample size) reference panel and C genotype panel

## Discussion

In this study, we proposed a new strategy for increasing imputation quality of rare variants, i.e., a combined approach that uses the genotype panel of combined data including SNP chip and exome chip for imputation. Using a WES + GWAS + EXOME reference panel, we showed that the genotype panel of combined data yielded better imputation quality than other genotype panels. For rare variants (MAF < 1 %), the combined approach relatively increased imputation quality up to 11 % and enhanced genomic coverage up to 117.7 %, as compared to the genotype panel of SNP chip only.

In addition, we systematically investigated the effect of various reference panels on imputation quality. We believe the current study is the first to systematically analyze imputation qualities by combining various reference panels and genotype panels. The best imputation quality for rare variants was obtained using the study-specific reference panel WES + GWAS + EXOME and the genotype panel of combined data.

Our study also provides a guideline for researchers to establish more cost-effective imputation strategies for increasing the imputation quality of rare variants. As shown in the results, combining a reference panel with a reasonable sample size and the combined data genotype panel is a cost-effective approach to increasing imputation quality of rare variants. For example, we reported that the genotype panel of combined data with 500 samples of reference panel outperformed the genotype panel of SNP chip with 848 samples of reference panel. The cost per sample of exome sequencing ($750) is about 11 times more expensive than those of exome chip ($70) [27]. If less than 3,700 samples were genotyped, generating exome chip data would be much more cost-effective than producing additional 348 samples of exome sequencing data. As an alternative to producing exome sequencing data or genotyping exome chip data, the merged panel approach which combine the concatenation of a public reference panel (e.g. 1,000 genomes project data) and a study specific reference panel, can be considered to increase the sample size of reference panel. Recent studies reported that the merged panel enhanced imputation performance [17, 28]. Also, 1,000 genomes project phase 3 data became available, providing 2,504 samples of whole genome sequencing data [29]. Since rare variants tend to be population specific [30], the merged panel approach would be effective if study samples were closely related with populations of 1KG data. Populations of 1KG phase 3 are African (661 samples), American (347 samples), East Asian (504 samples), European (503 samples), and South Asian (489 samples). Customized genotyping chip can also be an alternative approach if none of SNP chip and exome chip data is available. Considering the frequency of rare variant in a specific population, one can design a study specific genotyping chip containing rare variants. For example, UK biobank designed a chip containing 821 K SNPs [31]. Among them, about 111 K variants were rare coding variants (see url: http://www.ukbiobank.ac.uk/scientists-3/uk-biobank-axiom-array/). Therefore, based on our results, a rare variant association research considering the study data and populations of study samples can be designed more cost-effectively.

Despite providing a new imputation strategy and valuable insights for imputing rare variants, the current study has several limitations that should be noted. First, we removed substantial amount of variants from our analysis. Poorly-imputable variants (MAF < 0.3 %) were 43.1 % of variants in the initial reference panel. If additional true genotype data is available (e.g. exome sequencing data), dosage *r*^2^ can be calculated instead of estimated *r*^2^, then thorough analysis would be possible for all very rare variants. Second, the case samples of the reference panel may influence imputation results. Generally, public reference panels are consists of normal samples [8]. In this study, the 848 samples of the reference panel are consists of 415 type 2 diabetes and 433 controls. To analyze the impact of samples with disease status in the reference panel on imputation performance, we performed imputation on chromosome 1 of combined genotype panel using the reference panel consisting of 415 cases and the reference panel consisting of 433 controls. After that, we calculated the Pearson correlation coefficient between imputation qualities of two reference panels. The Pearson correlation coefficient was 0.96. Also Duan et al. previously reported that there was no loss of imputation quality using the reference panel consisting of samples with phenotypic extremes or disease status [17]. Thus, imputation results of our study may not be influenced by cases of the reference panel. Third, the comparison analysis of this study may not be exhaustive. We compared the imputation performance of the reference panels; however, 1KG phase 3 data and the merged panel approach were not included in the analysis. Further study is warranted to compare the reference panels and related approaches exhaustively. Lastly, our study did not report explicit cost benefits by imputation strategies considering costs of bioinformatics and whole genome sequencing. In practice, sequencing data analysis and imputation are not free of charge in considering compute-intensive analysis. Therefore, explicit cost-benefits analyses that argue for optimal designs in light of difference cost structures are required for a further study.

Next generation sequencing (NGS) provides base-pair resolution data and generated a near complete catalogue of genetic variants in human genome [8]. Unlike previous genome studies that focused on using initial genomic technologies such as chip-based genotyping on common variants, NGS is expected to uncover the role of less common and rare variants in various diseases. However, due to high cost and computation-intensive analysis, large-scale, population based genomic sequencing studies are not yet feasible [10, 11]. Although NGS is not yet cost-effective for large-scale genome studies, it will soon become essential, as its cost rapidly decreases. Meanwhile, imputation-based research strategies would be cost-effective for identifying associations between diseases and variants, including less common and rare variants.

## Conclusions

Here, we proposed a combined approach that imputes rare variants using the genotype panel of combined data including SNP chip and exome chip. We evaluated the performance of the combined approach using 848 samples from a study-specific reference panel and 5,349 samples of genotype panels consisting of exome chips only, SNP chips only, and combined data from an exome chip and a SNP chip. For rare variants (MAF < 1 %), the combined approach greatly increased imputation quality approximately 11 % compared to that of the exome chip only and showed up to a 117.7 % increase in genomic coverage. The proposed combined approach would be a cost-effective strategy to obtain better imputation quality and enhanced genomic coverage for rare variants.

We also systematically investigated the effect of various combinations of reference panels and genotype panels. The best performing approach combined data from a study-specific reference panel and a genotype panel.

## Methods

### Study samples

As part of the Korean Genome Analysis Project, Korea Association REsource (KARE) study was initiated in 2007 to conduct a large-scale genome-wide association study aiming to discover variants associated with Type 2 diabetes and numerous complex traits. The detailed information has been described elsewhere [32]. Briefly, a total of 10,038 participants aged 40 to 69 were recruited from two population-based cohorts comprising the Ansung (*n* = 5,018) and Ansan (5,020) cohorts. In this study, we used exome sequencing data and genotyping data from KARE samples. All participants of KARE provided written informed consent. The study using KARE samples was approved by two independent institutional review boards at Seoul National University and the National Institute of Health, Korea.

### Exome sequencing

By the Type 2 Diabetes Genetic Exploration by Next-generation Sequencing in Ethnic Samples (T2D-GENES) Consortium, about 10,000 exomes from five ethnic groups were sequenced using Agilent Human Exon v2 capture (~18,000 genes) at the Broad Sequencing Center. Among these, part of the samples are from the KARE project [32], including unrelated 538 type 2 diabetes samples and 579 control samples, were included, and 1,087 samples were used for further analysis after sample quality control. The reference genome hg19 was used for alignment and variant calling performed using the Genome Analysis Toolkit v2 [33]. Among 1,087 exome sequenced samples, only 848 samples had data of both SNP chip and exome chip. As a result, 500,821 autosomal variants from 848 Korean samples were used for constructing reference panels. The accuracy of the called variants was calculated by comparing genotypes from sequencing data with genotypes of genotyping chip data, showing overall concordances of 99.76 % and 99.96 % for the Affymetrix 5.0 and exome chips, respectively.

### SNP chip and exome chip data

Previously, 8,842 samples of KARE project were genotyped using the Affymetrix Genome-Wide Human SNP Array 5.0 (Affymetrix Inc., San Diego, CA, USA) [32]. Among them, 6,197 identical samples were genotyped using the Illumina HumanExome BeadChip v1.1 (Illumina, Inc., San Diego, CA, USA) exome chip. For the two platforms, standard sample quality controls were conducted, excluding those with a high missing rate (>4 %), gender discrepancy, excessive heterozygosity, or cryptic first-degree relatives. Exclusion criteria for the Affymetrix SNP chip were: Hardy-Weinberg equilibrium *p*-values < 10^−6^, genotype call rates < 95 %, and MAF < 0.01. All SNP chromosomal positions were updated to hg19 using the Affymetrix annotation file. Quality control of exome chip variants was similar to that of the SNP chip, except for the threshold for filtering out variants with low allele frequency. Only monomorphic variants were excluded for further analysis. From quality controlled data, we used 6,197 samples that were common between sets of the Affymetrix SNP chip and exome chips. Variants included in the analysis were 344,366, and 66,196 for the SNP chip and exome chip, respectively. Among 6,197 samples, 848 samples were used for constructing the reference panel, and the remaining 5,349 samples were used for genotype panels.

### Construction of reference panels

We constructed the reference panel by merging whole exome sequencing, exome chip, and SNP chip of 848 identical samples. Prior to merging, overlapping variants between the WES and chip data were removed from chip datasets. For overlapping variants between GWAS and EXOME, variants from EXOME were used to remove overlapping variants from GWAS. After merging all the data, the initial reference panel contained 856,690 variants. For comparisons, we excluded poorly imputable variants (MAFs < 0.3 %) for further analysis. The final WES + GWAS + EXOME reference panel contained 487,381 variants and was phased using the ShapeIT v2 program [34]. After phasing, a subset of variants from the WES + GWAS + EXOME reference panel was selected for constructing three reference panels: WES, WES + GWAS, and WES + EXOME. We also downloaded 1,000 genomes phase I Shapeit2 reference with no monomorphic and no singleton sites from MACH website (http://www.sph.umich.edu/csg/abecasis/MACH).

### Construction of genotype panels

Among 6,197 samples, 5,349 samples remained after excluding 848 samples were used for constructing the reference panel. The genotype panel consisting of exome chip of 5,349 samples was phased using the ShapeIT v2 progam. As the genotype panel of SNP chip only, SNP chip data of 5,349 samples were phased using the ShapeIT v2 program. For the genotype panel of combined data, the SNP chip and exome chip of 5,349 identical samples were merged and phased using the ShapeIT v2 program.

### Statistical analysis

In this study, we performed typical pre-phasing-based imputation on genotype panels [35]. For imputation, we used minimac software, a low memory and computationally efficient implementation of the MaCH algorithm [25]. The dosage *r*^2^ was accessed by calculating squared Pearson correlations between imputed dosages and true genotypes from exome chip. For comparison analysis of imputation performance, we used ${\widehat{r}}^2$ provided by minimac as an imputation quality measure. To compare the imputation results between pairs of genotype panels, Wilcoxon signed-rank tests were performed for ${\widehat{r}}^2$ values of imputed variants. Statistical analyses and visualization of the results were performed using the R program (http://www.r-project.org).

### Availability of data

Exome sequencing data will be available on dbGAP. The genotype data of KARE samples are available by sending a request to the Distribution desk of Korea Biobank Network, National Institute of Health, Korea.

## Abbreviations

GWAS: Genome-wide association study
MAF: Minor allele frequency
SNP: Single nucleotide polymorphism
WES: Whole exome sequencing data
EXOME: Exome chip data
1KG: 1,000 genomes phase 1 dataset
NGS: Next generation sequencing
KARE: Korea Association REsource
T2D-GENES: Type 2 Diabetes Genetic Exploration by Next-generation Sequencing in Ethnic Samples
